# Enhancing quality of life and medication adherence for people living with HIV: the impact of an information system

**DOI:** 10.1186/s41687-023-00680-x

**Published:** 2024-01-23

**Authors:** Maria Panayi, Georgios K. Charalambous, Eleni Jelastopulu

**Affiliations:** 1 Ph.D. Programme Health Management, Frederick University, Nicosia, Cyprus; 2 Gregorios AIDS Clinic, General Hospital of Larnaca, Larnaca, Cyprus; 3 Hippocration General Hospital, Athens, Greece; 4 Department of Public Health, School of Medicine, University of Patras, Patras, Greece

**Keywords:** Adherence, Compliance, Quality of life, HIV/AIDS, Mobile health

## Abstract

**Background:**

The widespread availability of antiretroviral therapy has led to improvements in life expectancy and thus an increase in the number of people living with HIV/AIDS (PLWHA) worldwide. However, a similar increase in the number of newly-diagnosed patients in Cyprus suggests the need for solutions designed to improve monitoring, planning, and patient communication. In this study, we aimed to determine whether the use of an information system to manage PLWHA might contribute to improved quality of life and critical adherence to prescribed drug regimens and ongoing medical care.

**Methods:**

A randomized controlled trial study was conducted in Cyprus based on information that we collected using the highly valid and reliable Greek translation of the World Health Organization (WHO) Quality of Life (QOL) HIV-BREF questionnaire to assess sociodemographic variables and patient compliance. We distributed 200 questionnaires before implementing a Health Medical Care (HMC) information system at our clinic. Six months after implementing this system, 68 of the completed questionnaires were selected, including two groups of 34 participants who had been assigned at random to the intervention or the control group. Participants included PLWHA aged ≥ 18 years who had been receiving antiretroviral therapy for more than 12 months between July 15, 2020, and July 15, 2022.

**Results:**

The changes in baseline to six-month scores reported for the intervention group were significantly higher than in the control group in all six subscales assessed with the WHOQOL-HIV-BREF questionnaire, as well as in the assessment of compliance. Furthermore, compliance with treatment was associated with higher scores in the questionnaire subscales, including physical health, psychological health, degree of autonomy, social relationships, life circumstances, and spirituality/religious/personal beliefs. We also identified specific demographic factors and behaviors that were associated with better compliance with scheduled medical care and the prescribed drug regimen. Specifically, men exhibited better compliance than women and younger PLWHA exhibited better compliance than the elderly as did individuals who reported a higher level of educational attainment. Additionally, individuals who did not use addictive substances, consumed less alcohol, and were managed using the monitoring information system all exhibited better compliance compared to those in the control group.

**Conclusion:**

The results of this study suggest that management of PLWHA via the use of an information system can contribute to improved QOL and drug compliance.

## Abstract (plain language summary)

This study was conducted to find ways to improve the lives of people living with HIV/AIDS (PLWHA) in Cyprus. With more PLWHA receiving treatment and care, it's crucial to ensure they have a good quality of life and follow their treatment plans. The main question we wanted to answer was whether using a special computer system to manage PLWHA could make their lives better and help them stick to their treatment. We found that using this computer system indeed made a positive difference. People who used it reported better physical and mental health, more independence, better relationships, and overall improved life circumstances. The study showed that managing PLWHA with a computer system can lead to better quality of life and treatment adherence. Some groups, like men, younger individuals, and those with more education, did even better. Also, people who didn't use addictive substances or consume much alcohol had better results. In summary, using technology in healthcare can greatly benefit PLWHA in Cyprus. This study highlights the importance of innovative solutions in healthcare to enhance the well-being of people living with HIV/AIDS and ensure they receive the best possible care.

## Background

In light of extraordinary medical progress in the past several decades, several diseases that were once considered fatal have now become chronic conditions. This is clearly the case for people living with HIV/AIDS (PLWHA). With proper adherence to medication, PLWHA can experience prolonged life expectancy and a reasonable quality of life (QOL). According to the statistics from the United Nations for 2020, there are 37.7 million PLWHA worldwide, including 1.7 million children aged 0–14 years. Of note, 53% percent of the PLWHA are females. While 83% of all PLWHA are aware of their HIV-positive status, 6.1 million people worldwide remain unaware of their diagnosis. Furthermore, HIV/AIDS-related deaths have decreased by 64% since 2004 and by 47% since 2010. Only 680,000 HIV-associated deaths were reported in 2020, representing a significant decline in HIV-related mortality compared to what was observed in previous years. These positive developments have been attributed to the availability of effective antiretroviral therapy (ART) and critical patient education. While only 7.8 million PLWHA had access to treatment in 2010, by 2020, this number had increased to 27.5 million, with 74% of adults aged 15 years and older having access to ART. Additionally, a full 85% of pregnant women diagnosed with HIV had access to ART and could thus prevent transmission of the virus to their unborn children [1].

Nowadays, medical professionals globally are focused on achieving the WHO’s ambitious goal, known as the "95%–95%–95%" target. This goal encompasses three key objectives designed to ensure that (i) 95% of the HIV-infected population is diagnosed, (ii) 95% of diagnosed individuals initiate antiretroviral therapy (ART), and (iii) 95% of those on ART achieve the desired outcome of an undetectable viral load [2]. As of 2020, among PLWHA who were aware of their HIV status, 87% had access to treatment, and 90% successfully achieved virus suppression. More specifically, among all PLWHA, 73% had initiated treatment and 66% had achieved viral suppression [1]. Unfortunately, the number of new HIV/AIDS cases continues to increase across the globe [3]. Thus, all countries must determine how to respond effectively to the growing demand for long-term care. Any decision or collective efforts directed at providing care for PLWHA should prioritize and strive for optimal QOL.

**Fig. 1:**
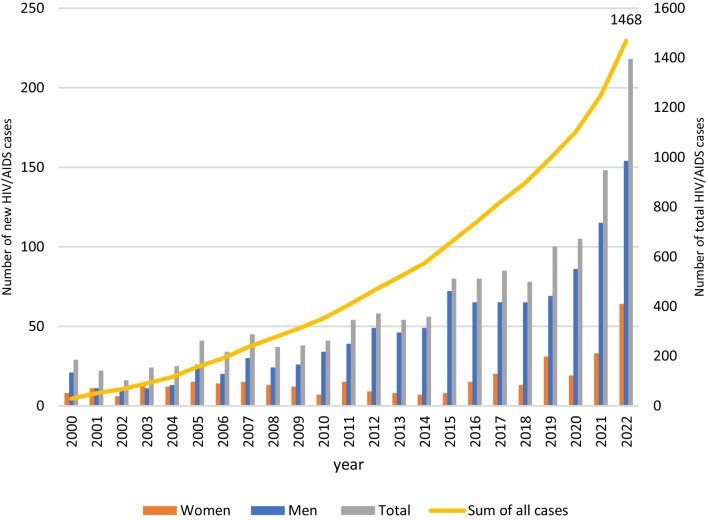
Total number of new HIV/AIDS cases in Cyprus per year and gender and total prevalent cases, 2000–2022 (Epidemiological Bulletin of the Ministry of Health, Cyprus)

According to the new registrations of HIV-positive people in the system of the Republic of Cyprus, in the last ten years the registrations of people living with HIV have more than doubled. Specifically, from 1986 to 2010, 681 people with HIV were monitored, while from 2011 to 2021, the number of new registrations of people monitored at the Gregorios Clinic of Larnaca General Hospital, the only HIV National Reference center in Cyprus, amounts to 900 (Fig. 1) [4]. If we compare the last two decades, we can see that from 2000–2010 new registrations amounted to 274 while from 2011 to 2021 they have almost tripled. One of the most important problems in the management of these individuals is the constant increase in new cases (Fig. 1) [4]. This problem, combined with shortages in the medical and nursing staff and the increase of comorbidities due to long life expectancy [2, 4], makes proper and effective follow-up check of people living with HIV in Cyprus even more difficult.

The objective of this study was to determine whether managing PLWHA using a Health Medical Care (HMC) information system could have a positive impact on patient QOL and compliance. Our implementation and evaluation of a newly-established information system will permit new goals to be identified and established and may lead to improved monitoring, treatment planning, and ultimately a better QOL for PLWHA in Cyprus.

## Methods

### Study design-setting

This randomized controlled trial (RCT) study was conducted at the Gregorios Clinic of the Lanarca General Hospital, which is the HIV National Reference clinic in Cyprus. Data collection took place between July 15, 2020 and July 15, 2022. Participants were selected randomly and completed the questionnaires during their routine visits to the Gregorios Clinic of Larnaca General Hospital.

### Participants

The participants of this study were PLWHA who were monitored at the Gregorios Clinic of Larnaca General Hospital. Patients were included if they fulfilled the following criteria: (1) ≥ 18 years of age; (2) actively monitored at the Gregorios Clinic, indicating a confirmed diagnosis of HIV; and (3) had received antiretroviral treatment for a minimum of one year so that adherence could be measured before and after implementation of the information system (HMC).

### Measures and instruments

Participants were asked to complete a structured questionnaire that was designed by the researchers to collect information focused on socioeconomic status, clinical condition, and compliance with their prescribed care and medication regimen. The questionnaire collected information on various socioeconomic characteristics, including age, gender, sexual identity, marital status, level of educational attainment, annual income in the previous year, and substance use, including addictive substances and alcohol. Ten self-developed questions were introduced to measure the participants’ compliance. These questions were designed to evaluate disease management, specifically, adherence to medication and scheduled appointments for laboratory tests. Each participant received an overall compliance score that ranged from 0 to 28. Participants with scores of 0 to 3 were classified as having good compliance. Those with scores of 4 to 6 were identified as moderately compliant, while scores of 7 or higher indicated poor compliance.

We also asked the participants to complete the WHOQOL-HIV BREF questionnaire at the same time.

This questionnaire was developed by the World Health Organization (WHO) to measure the Health-Related Quality of Life (HRQOL) of HIV/AIDS patients. This questionnaire includes 31 items that cover six domains, including physical health (four items), psychological health (four items), level of independence (four items), social relationships (four items), environment (eight items), and spirituality (four items), along with two additional general items. The summary domain and total HRQOL scores were calculated using the scoring method developed by the WHOQOL-HIV group [5]. The researchers ensured the anonymity of the questionnaire by sealing it in an envelope. Upon receipt, they promptly coded the questionnaires to safeguard participant identities. Participants were randomly selected during their routine visits to the Grigorio Clinic at Larnaca General Hospital, minimizing potential bias.

Two hundred participants responded to the questionnaire at the study's outset. Subsequently, a sample of 34 patients was chosen for the six-month pilot implementation of the HMC information system. Subsequently, a sample of 34 patients was selected for the six-month pilot implementation of the HMC information system. The questionnaire was once again administered to this patient cohort, comprising the intervention group. Simultaneously, another group of 34 individuals without access to the information system completed the questionnaire, constituting the control group. To ensure data matching before and after the intervention, sociodemographic information was collected for each participant.

**Fig. 2:**
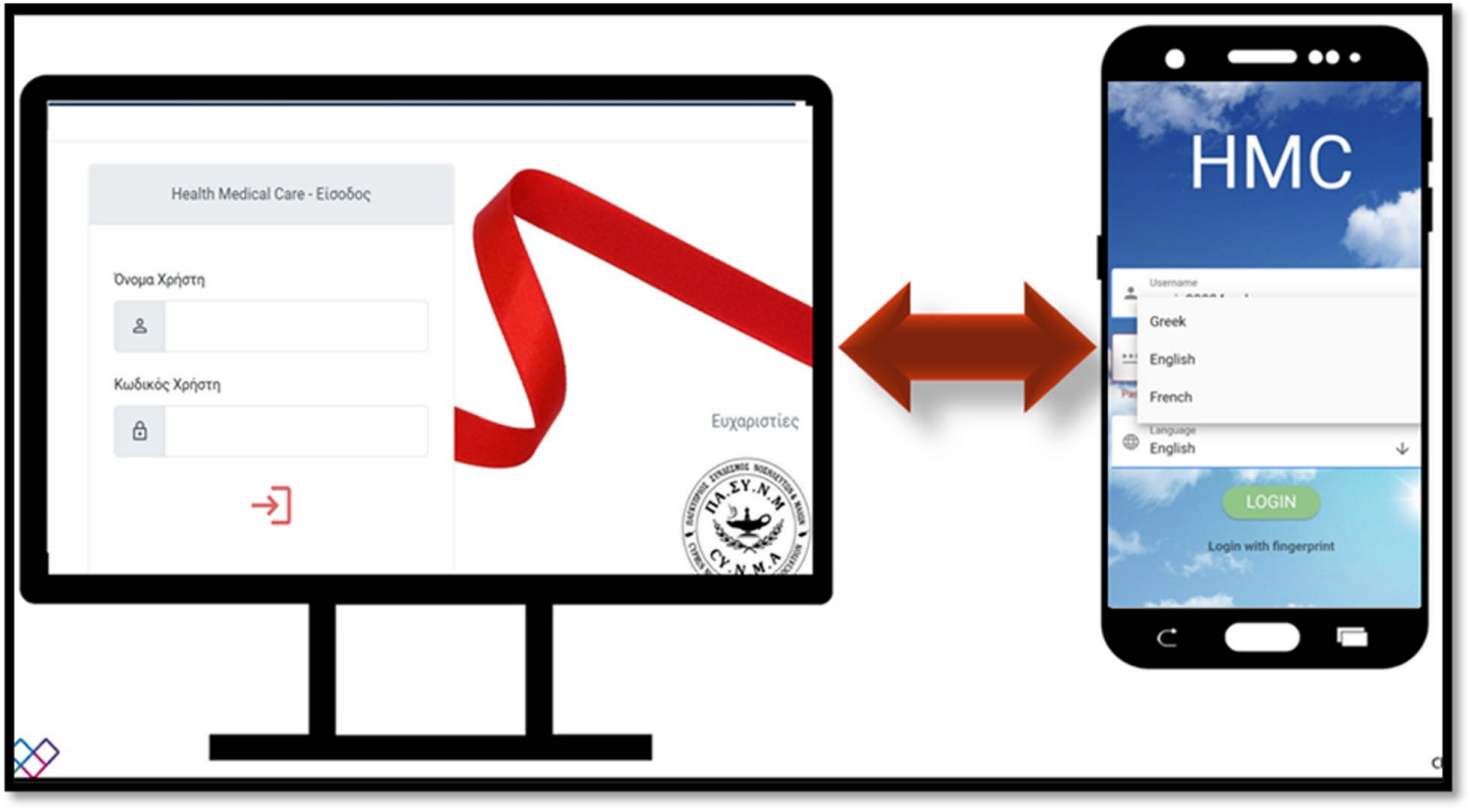
Initial page of the developed Android and Web application (the Web application, mainly for the medical and nursing staff, is presented on the computer screen and the Android application, mainly for the PLWHA, is presented on the mobile screen)

The HMC is an information system consisting of a Web Application (for medical and nursing staff) and an Android Application (for PLWH) where they interact in real time and aim to ensure the quality of life of HIV-positive people in Cyprus (Fig. 2). Specifically, the Android Application helps to improve the self-monitoring of PLWH, through access to the results of their analyses, timely information about appointments, information regarding medical news around HIV and the Gregory Clinic, solving questions, sending messages with real-time response, etc. While the Web application allows medical and nursing staff to better track laboratory results, compliance with scheduled laboratory test appointments, prescription renewal and access to the patient's vaccination card. In addition, scheduling is automated according to international protocols for both HIV and syphilis, enabling medical and nursing staff to have two-way communication with each PLWH in real time. Moreover, the doctor can enter the prescription and the medical history (including the admission file). It is important to note that the app does not provide any information, including the phone number, email, Facebook account, messenger, or any details that would identify the person coded into the app. Both the Web App and the Android App require passwords for access, and each HIV-positive individual has exclusive access to their personal account. No HIV-positive individual is able to access another HIV-positive account, even if they know the password.

### Bias

To ensure impartiality, specific criteria for the exclusion of participants were established at the beginning of the study to minimize bias. Individuals aged ≤ 18 years, not actively monitored at the Gregory Clinic and not receiving antiretroviral therapy for at least one year were specifically excluded.

### Study size

Convenience sampling was considered the most suitable approach for data collection in this study, as it enabled us to include as many willing participants as possible more easily, quickly, and at a low cost, during a period of movement restrictions [6].

To meet the minimum sample requirements for all assessments, we distributed questionnaires to 200 patients before implementing the information system. Six months after the implementation of this system, 68 questionnaires were collected (34 from the implementation group and 34 from the controls), following a random selection from the original pool of 200 participants.

### Statistical analysis

For descriptive statistical analysis, continuous variables were presented as mean values (mean, M) with calculated standard deviation (SD). Discrete variables were reported as frequencies (N) and relative frequencies (N%). The normality assumption was assessed using the Shapiro–Wilk test, as well as graphical representations, including the normal Q–Q plot, the detrended normal Q–Q plot, and the box plot.

The Wilcoxon signed-rank test was used to assess antiretroviral adherence outcomes in treated and untreated subjects with the HMC information system. To test the correlations between WHOQOL-HIV-BREF subscale scores and adherence level we used Kendall's tau_b.

Statistical analysis was performed using SPSS version 25 and STATA version 12 software. The minimum level of statistical significance was set at 5%. Analysis of Gain Scores were used to determine whether the intervention group exhibited significantly different pre-test to post-test scores compared to the control group.

## Results

### Sample characteristics

The initial study population included 200 HIV-positive patients (PLWHA) who were monitored at the Gregorios Clinic of Larnaca General Hospital in Cyprus. The mean time since diagnosis was 9.9 years (standard deviation [SD], 7.8 years). As shown in Table 1, the initial participant group included 142 males (71%) and 58 females (29%). Of this cohort, 14.5% were 18–30 years of age, 36% were 31–40 years of age, 22% were 41–50 years of age, and 27.5% were > 50 years old. Seventy percent of these participants reported having a secondary education, while 18% had a primary education only and 12% reported tertiary education. Fifty-four percent of the participants were single, 24% were married, 15% were divorced, and 7% were widowed. Similarly, 28% of patients reported having no income while 16% reported income levels < €10,000 per year. Others (18.5%) reported an annual income between €10,001 and €15,000 per year. 18.5% of these people reported an annual income between 15,001 and 20,000 euros. While another 13% reported an annual income between €20,001 and €30,000, only 8.5% reported an income exceeding €30,000 per year. This patient cohort included 78% Greek natives and 22% who were foreigners in Cyprus. Furthermore, 25.5% of participants reported illicit use of addictive substances. While 60.5% of the participants reported that they never consumed alcohol, of those who did, 17% reported that they did so only on rare occasions, 16.5% consumed alcohol weekly, and 6% consumed alcohol on a daily basis.

**Table 1:** Participant characteristics (N = 200)

	N	%
Sex		
Men	142	71.0
Women	58	29.0
Age (years)		
18–30	29	14.5
31–40	72	36.0
41–50	44	22.0
> 50	55	27.5
Sexual orientation		
Heterosexual	74	37.0
Homosexual	94	47.0
Bisexual	26	13.0
Transgender	1	0.5
Other	5	2.5
Educational level		
Primary education	36	18.0
Secondary education	140	70.0
Higher education	24	12.0
Marital status		
Married	48	24.0
Divorced	30	15.0
Widowed	14	7.0
Unmarried	108	54.0
Annual income		
No income	32	16.0
< 10,000 €	56	28.0
10,001–15,000 €	32	16.0
15,001–20,000 €	37	18.5
20,001–30,000 €	26	13.0
> 30,000 €	17	8.5
Nationality		
Greek	156	78.0
Other	44	22.0
Use of addictive substances		
No	149	74.5
Yes	51	25.5
Alcohol consumption		
Never	121	60.5
Rarely	34	17.0
Weekly	33	16.5
Daily	12	6.0
Comorbidities		
No	117	58.5
Yes	83	41.5
Do you know the medication you are taking?		
No	92	46.0
Yes	108	54.0
Does the treatment you are receiving seem to be helping you?		
No	12	6.0
Yes	188	94.0

Finally, we note that 41.5% of reported comorbidities, and 54% reported that they were aware of the names of the medications they were taking. Most (94%) believed that the treatments that they were receiving were beneficial (Table 1).

### Impact of the implementation of an information system on quality of life

**Fig. 3:**
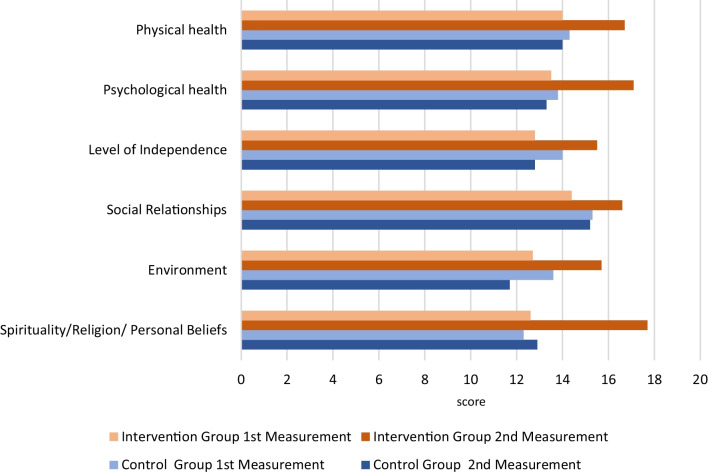
Perceptions of Quality of Life at Baseline Measurement and after six-month-follow-up by patient group

To determine whether the implementation of the monitoring system had a significant impact on patient QOL, we examined changes from baseline WHOQOL-HIV-BREF subscale scores reported by participants who were managed for six months using this system compared to those from patients who received routine care. Of note, we observed no statistically significant differences in the mean baseline scores in the intervention compared to the control group. The mean (M) ± standard deviation (SD) of the baseline scores on the physical health subscale were 12.6 ± 3.5 and 12.3 ± 5 in the intervention and control groups, respectively, with t(67) = 0.292 and *p* = 0.771. The M ± SD of the baseline scores on the psychological health subscale were 12.7 ± 4.3 and 13.6 ± 4.2 in the intervention and control groups, respectively, with t(67) = −0.892 and *p* = 0.375. The M ± SD of the baseline scores on the degree of autonomy subscale were 14.4 ± 4.1 and 15.3 ± 5.3 in the intervention and control groups, respectively, with t(67) = −0.759 and *p* = 0.451. The M ± SD of the baseline scores on the social relations subscale were 12.8 ± 4.6 and 14 ± 4.5 in the intervention and control groups, respectively, with t(67) = −1.104 and *p* = 0.274. The M ± SD of the baseline scores on the living conditions subscale were 13.5 ± 3.6 and 13.8 ± 3.9 in the intervention and control groups, respectively, with t(67) = −0.315 and *p* = 0.754. Finally, the M ± SD of the baseline scores on the spirituality/religiosity/personal beliefs subscale were 14 ± 4.2 and 14.3 ± 4.4 in the intervention and control groups, respectively, with t(67) = −0.276 and *p* = 0.783 (Table 2; Fig. 3).

**Table 2:** Mean subscale scores on the two measures by patient group

Group	WHOQOL-HIV-BREF subscales	1st measurement		2nd measurement	
		Mean	SD	Mean	SD
Intervention (n = 34)	Physical health	12.6	3.5	17.7	1.6
	Psychological health	12.7	4.3	15.7	2.9
	Degree of autonomy	14.4	4.1	16.6	3.3
	Social relationships	12.8	4.6	15.5	3.5
	Life conditions	13.5	3.6	17.1	1.4
	Spirituality/religious/personal Beliefs	14.0	4.2	16.7	3.6
Control (n = 34)	Physical health	12.3	5.0	12.9	4.7
	Psychological health	13.6	4.2	11.7	4.4
	Degree of autonomy	15.3	5.3	15.2	4.8
	Social relationships	14.0	4.5	12.8	5.1
	Life conditions	13.8	3.9	13.3	4.1
	Spirituality/religious/personal beliefs	14.3	4.4	14.0	4.6

### Comparisons between the intervention and control groups

The mean changes in the scores (baseline to six-month follow-up) exhibited by participants in the intervention and control groups were assessed for each subscale of the WHOQOL-HIV-BREF using the Analysis of Gain Scores and independent samples *t*-tests based on the assumption that the data were normally distributed. The results of the independent samples *t*-tests revealed that the differences in baseline-to-six-month scores reported for the intervention group were significantly different from those reported for participants in the control group across all six subscales assessed by the WHOQOL-HIV-BREF questionnaire. Specifically, participants managed with the HMC information system responded with significant improvements in all six categories, including physical health, psychological health, degree of autonomy, social relations, living conditions, and spirituality/religion/personal beliefs.

Mean differences (assessed by subtracting the baseline from the six-month measurement for each subscale) were determined for participants in the intervention and the control groups. The results revealed several statistically significant differences between the two groups. We found that participants in the intervention group exhibited significantly higher mean score differences (M ± SD) on the physical health subscale compared to those in the control group, at 5.1 ± 3 *versus* 0.6 ± 6.7, respectively, with t(46,234) = 3.564 and *p* = 0.001. Participants in the intervention group also exhibited significantly higher mean score differences on the psychological health subscale compared to those in the control group, at 3 ± 3.3 *versus* − 2.1 ± 5, respectively, with t(66) = 4.993 and *p* < 0.001. Similar results were observed for other subscales. For example, participants in the intervention group exhibited significantly higher mean score differences on the degree of autonomy subscale compared to those in the control group, at 2.2 ± 4.7 *versus* − 0.4 ± 5.3, respectively, with t(66) = 2.113 and *p* = 0.038. Participants in the intervention group exhibited significantly higher mean score differences on the social relations subscale compared to those in the control group, at 2.7 ± 5.4 *versus* − 1.1 ± 6.1, respectively, with t(66) = 2.749 and *p* = 0.008. Similarly, participants in the intervention group exhibited significantly higher mean score differences on the life conditions subscale compared to those in the control group, at 3.6 ± 3.4 *versus* − 0.7 ± 4.9, respectively, with t(66) = 4.152 and *p* < 0.001. Finally, participants in the intervention group exhibited significantly higher mean score differences on the spirituality/religious/personal beliefs subscale compared to those in the control group, at 2.8 ± 4.4 *versus* − 0.2 ± 5.5, respectively, with t(66) = 2.456 and *p* = 0.017. Table 2 provides the mean scores for the two measurements, along with the corresponding standard deviations, for each group of patients (intervention and control).

### Adherence to antiretroviral therapy and quality of life

**Fig. 4:**
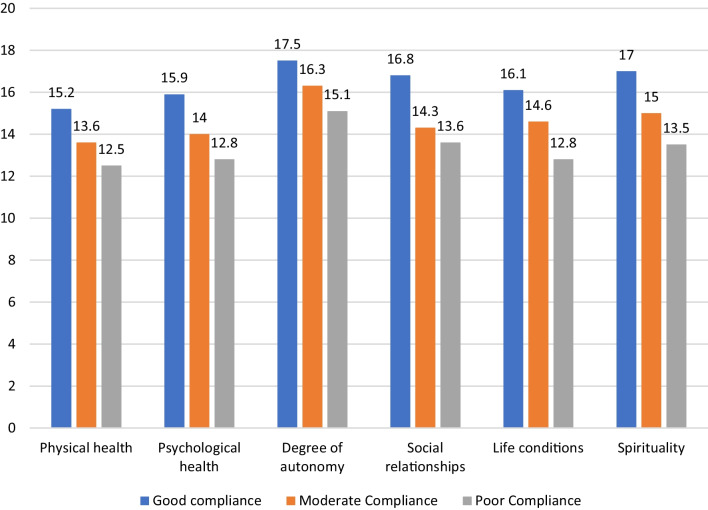
Correlation between the quality of life and adherence to antiretroviral treatment

As shown in Table 3, we found that higher WHOQOL-HIV-BREF scores overall were associated with better patient adherence across all subscales. Specifically, a detailed analysis of the results shown in Table 3 revealed negative significant associations between subscale scores and patient adherence. For example, our findings revealed a statistically significant negative relationship between scores on the physical health subscale and the degree of medical compliance (τb(200) = −0.177, p = 0.002) or on the psychological health subscale and the degree of compliance (τb(200) = −0.262, p < 0.001). Furthermore, our findings revealed a statistically significant negative correlation between the scores on the degree of autonomy subscale and the degree of compliance (τb(200) = −0.208, p = 0.001), on the social relations subscale and the level of compliance (τb(200) = −0.264, p < 0.001), on the life conditions subscale and the degree of compliance (τb(200) = −0.224, p < 0.001), and on the spirituality/religious/personal beliefs subscale (τb(200) = −0.262, p < 0.001) that correlate with better adherence. This result suggests that higher scores on the physical health, the psychological health, on the degree of autonomy, on the social relations, on the life conditions, and on the spirituality/religious/personal beliefs subscale are linked to better adherence (Table 3). Figure 4 further illustrates the correlation between the degree of adherence to antiretroviral treatment and the quality of life. Notably, the data indicate that higher scores across all subscales are associated with better treatment compliance.

**Table 3:** Tests of correlations between WHOQOL-HIV-BREF subscale scores and adherence level (N = 200)

*Kendall's tau_b*	
Physical health	
Correlation coefficient	− .177**
Sig. (2-tailed)	.002
Psychological health	
Correlation coefficient	− .262**
Sig. (2-tailed)	.000
Degree of autonomy	
Correlation coefficient	− .208**
Sig. (2-tailed)	.001
Social relationships	
Correlation coefficient	− .264**
Sig. (2-tailed)	.000
Life conditions	
Correlation coefficient	− .224**
Sig. (2-tailed)	.000
Spirituality/religious/personal beliefs	
Correlation coefficient	− .262**
Sig. (2-tailed)	.000
N	200

### Adherence to antiretroviral therapy in individuals who were and were not managed with a monitoring system

The Wilcoxon signed-rank test revealed a statistically significant improvement in medical adherence (z = −3.819, *p* < 0.001) for those managed with a monitoring system. By contrast, no statistically significant improvements in adherence were observed (z = −1.414, *p* = 0.157) in subjects who did not have the monitoring system in place (Table 4). Interestingly, no significant associations were found between compliance and marital status, economic status, ethnicity, years since diagnosis, or specific comorbidities.

**Table 4:** Allocation of patients by group and measurement

Group	Degree of compliance (1st measurement)			Degree of compliance (2nd measurement)		
	Good compliance	Moderate compliance	Poor compliance	Good compliance	Moderate compliance	Poor compliance
*Intervention*						
Count	13	14	7	26	6	2
Row N %	38.2%	41.2%	20.6%	76.5%	17.6%	5.9%
*Control*						
Count	18	4	12	18	6	10
Row N %	52.9%	11.8%	35.3%	52.9%	17.6%	29.4%

## Discussion

The association between adherence to ART and improved QOL has been demonstrated consistently in previous research studies [7–19]. This finding was further supported by the findings presented in this study that revealed a positive correlation between improved compliance and higher scores in various domains of QOL, including physical health, psychological health, degree of autonomy, social relations, life conditions, and spirituality/religious/personal beliefs.

Our findings can be compared to those reported by Silva et al. [7], who found that PLWHA in Brazil who did not adhere to the ART regimen received the lowest scores in all domains of QOL. Among their conclusions, these researchers identified ART adherence as a positive factor related to QOL because of its beneficial effects on immunity, control of viral load, and disease progression.

It is critical to emphasize that achieving virus suppression and reducing viral load in HIV-infected individuals requires not only strong adherence to ART but to appropriate medical treatment [8, 20–23]. Results from several previous studies revealed that PLWHA who receive appropriate medical treatment will have a life expectancy similar to that experienced by the general population [24, 25]. However, achieving this near-normal life expectancy is contingent upon at least 95% adherence to ART to prevent treatment failure and the development of drug resistance [8, 26, 27]. Thus, the evaluation of new therapeutic options and technologies aimed at improving healthcare for PLWHA should include assessments of HRQOL in addition to clinical endpoints such as CD4^+^ T-cell count, viral load, and disease progression [24].

A research study conducted in Baghdad by Imam et al. [27] highlighted a strong relationship between adherence to a highly-active ART (HAART) regimen and quality of life (QOL). The findings presented in this study revealed that patients with only low-to-moderate adherence to HAART were 60% less likely to present with high global QOL scores compared to patients with high adherence to this regimen. As stated by Tsiadis [28], p. 33], "poor adherence leads to significant morbidity, mortality, and avoidable health care costs." He also noted that while technology can be a valuable tool in improving patient compliance, these devices are not stand-alone solutions to this problem.

Mobile devices, particularly those capable of transmitting and receiving short message service (SMS) communications (i.e., texts), have become increasingly prevalent in HIV/AIDS clinics as a means to support interactions with patients. In 2010, Lester et al. [29] conducted the first randomized clinical trial that explored the use of cellular devices to monitor the adherence of HIV-positive patients to first-line ART. The results from this study revealed that the use of cellular devices led to a modest improvement in self-reported adherence and viral suppression. Likewise, results from a study published by Luque et al. [30] revealed a significant increase in self-efficacy among study participants who utilized the personal health record application.

In recent years, there has been a notable rise in the development and implementation of mobile health (mHealth)-based self-management interventions for PLWHA. These interventions were demonstrated to be feasible, acceptable, and effective when used by study participants [24]. As stated by Muessig [31], p. 15], recent mHealth interventional tools designed to support medication adherence "show acceptability, feasibility, and preliminary efficacy in increasing ART use intentions, self-reported adherence, and viral suppression". Similarly, in a literature review published by Cooper et al. [24], the authors concluded that mHealth interventions can have a significant impact on various outcomes related to HIV care. These outcomes include ART adherence [32–39], suppression of viral load [34, 37, 38], engagement with care [40], HIV knowledge, depression, anxiety, and self-efficacy [31].

Many mHealth interventions provide patients with a wide range of functionality. One key aspect is their ability to monitor clinical indicators that reflect the symptoms and progression of the disease.

Furthermore, mHealth interventions facilitate self-management by enabling patients to monitor and control their diet and exercise routines and provide access to information that is tailored to specific guidelines and recommendations for managing HIV. Patients can also receive educational messages and electronic support from their peers that address their unique needs and preferences [24]. Many mHealth interventions also offer a high degree of flexibility and facilitate the delivery of customized content that can be tailored to the specific needs of each user [41]. The accessibility of mHealth interventions helps patients to overcome various social and structural barriers [42]. Furthermore, mHealth interventions have demonstrated great potential for scalability and diffusion across different geographic locations and may be particularly useful in resource-constrained settings [43].

The present study demonstrated that the implementation of an Android-application-based monitoring system led to improved adherence among those who participated in the intervention compared to those who did not. Additionally, we found that the implementation of this information system had positive effects on physical health, mental health, degree of autonomy, social relationships, and living conditions. These findings align with results published by Mehraeen et al. [43] which also highlighted the significant positive impact of mHealth strategies on disease prevention, ART adherence, and treatment for PLWHA. Furthermore, mHealth interventions had positive effects on behavioral and social problems that typically affect PLWHA. While the mHealth market needs to be regulated, we recognize the overall importance of mHealth for self-monitoring, self-care, and self-management by PLWHA [43].

However, despite the demonstrated benefits of mHealth interventions, there is a significant delay in their application in the clinical field, particularly in Cyprus. One of the major challenges faced by healthcare providers worldwide is securing the necessary financial resources to support the implementation of these critical interventions.

## Conclusions

The findings presented in this study highlight the positive impact of an HMC information system on QOL and medical adherence of PLWHA. The implementation of this system resulted in improvements in physical health, mental health, degree of autonomy, social relationships, and living conditions. Compliance with medical care and drug therapy were also positively associated with higher scores in various subscales on the WHOQOL-HIV-BREF questionnaire, including physical health, psychological health, degree of autonomy, social relationships, life circumstances, and spirituality/religious/personal beliefs. Our study also identified specific demographic factors associated with increased compliance. Male patients, those who are elderly, those with higher levels of educational attainment, individuals who do not use addictive substances, and those who utilized the monitoring information system were significantly more compliant with medical care and drug therapy. Overall, our findings indicate that managing PLWHA with an information system can contribute significantly to an improved QOL as well as adherence to life-saving medical treatment.

## Data Availability

The dataset used and analyzed in this study is available from the corresponding author upon reasonable request.

## Abbreviations

HMC: Health Medical Care
ART: Antiretroviral therapy
HIV: Human immunodeficiency virus
AIDS: Acquired immune deficiency syndrome
WHO: World Health Organization
PLWHA: People living with HIV/AIDS
